# Effects of Feed per Tooth and Radial Depth of Cut on Amplitude Parameters and Power Spectral Density of a Machined Surface

**DOI:** 10.3390/ma13061323

**Published:** 2020-03-14

**Authors:** Qing Zhang, Song Zhang

**Affiliations:** 1 Key Laboratory of High Efficiency and Clean Mechanical Manufacture of MOE, School of Mechanical Engineering, Shandong University, Jinan 250061, China; zhangqing302@163.com; 2 Key National Demonstration Center for Experimental Mechanical Engineering Education, Shandong University, Jinan 250061, China

**Keywords:** surface roughness, power spectral density, feed per tooth, radial depth of cut

## Abstract

Surface topography and roughness significantly affect the functional properties of engineering parts. In this study, a mathematical model simulating the surface topography in end milling is presented and verified by milling experiments. The three dimensional (3D) surface amplitude parameters (arithmetic average deviation *S_ba_* and root mean square deviation *S_q_*) of the milled surface were calculated by using the model and the effects of the product (*p*) and ratio (*r*) of radial depth of cut *a_e_* and feed per tooth *f_z_* on amplitude parameters were researched. To evaluate the lateral characteristics of the milled surface, one dimensional (1D) power spectral densities (PSD) along both feed and step-over direction were calculated and investigated. It was found that *f_z_* affects 1D PSD along both directions, whereas *a_e_* affects 1D PSD along the step-over direction. An angular spectrum, derived from the area power spectral density (APSD), was employed to research the spatial distribution of spectral energy on the milled surface. Furthermore, the influences of *p* and *r* on the PSD properties were researched. It was found that *r* is the significant factor that influences the direction of surface energy spectrum distribution.

## 1. Introduction

Surface roughness parameters that evaluate a machined surface can be classified into five categories: amplitude parameters, frequency parameters, hybrid parameters, functions and related parameters among others [1]. In the die casting process, friction is inevitable and surface topography of the dies significantly affects the interface friction behavior [2]. Root mean square (RMS) deviation of surface (*S_q_*), density of summits (*S_ds_*), and texture direction (*S_td_*) have been accounted to influence the frictional property of the surfaces [3]. Among which, *S_td_* is quantified by the angular spectrum of the surface which is derived from the area power spectral density (APSD). Besides, fatigue is another main failure mode of dies. The amplitude parameters of the machined surface have been proved to significantly affect the fatigue life of dies [4,5]. However, it has been suggested that *S_td_* has also a significant effect on fatigue strength [6]. In consequence, the amplitude parameters and power spectral density (PSD) have significant influences on the friction property and fatigue performance of dies. Therefore, it becomes an important factor for improving the service life of dies to investigate and control the factors that affect machined amplitude parameters and PSD.

The amplitude parameters describe the ups and downs of the machined surface profile among which arithmetic average deviation *S_ba_*, root mean square deviation *S_q_*, and 10-point surface height *S_z_* are most frequently used. In order to control the amplitude parameters, investigations on the effects of tool eccentricity and helix angle [7], tool run-out and deflection [8], cutter path [9], tool inclination [10], machining and cooling method [11,12], material hardness [13], and cutting parameters [14,15,16] on surface roughness have been conducted. Cutting parameters play an important role among the factors that affect surface roughness since they affect both the surface roughness and the machining efficiency which can be estimated by the material removal rate (MRR). According to Zhang et al. [17], MRR shows increasing tendency when the product (*p*) of radial depth of cut (*a_e_*) and feed per tooth (*f_z_*) increases. However, different combinations of *f_z_* and *a_e_* cause different surface topographies with a determined value of *p*. Therefore, in this paper, the ratio (*r*) of *f_z_* and *a_e_* is introduced to calculate the value of *f_z_* and *a_e_* when *p* is determined.

The amplitude parameters contain the information that is perpendicular to the machined surface, whereas the lateral information cannot be presented. In order to obtain the lateral and frequency domain properties of a machined surface, PSD has been used to analyze surface topography. The method is based on Fourier Transformation which treats signals as a combination of sinusoidal harmonics with different phases, amplitudes, and frequencies. PSD was first employed to specify the surface topography of optical surfaces [18,19]. It has now been gradually applied to the characterization and analysis of surface topography. Michalski [20] found it useful to use an angular diagram and contour map of PSD for texture direction estimation of gear teeth flank surface topography. Jacobs et al. [21] presented three important drawbacks that impede the application of PSD to the functional characterization of surface topography and proposed strategies to mitigate them. Krolczyk et al. [22] studied the effect of feed value, cutting tool vibration, and cutting tool wear on the surface texture of duplex stainless steel using power spectral analysis. The wavelengths were also compared for dry and minimal quantity cooling lubrication (MQCL) machining. Kubiak et al. [23] investigated the effect of initial roughness on the surface during friction and wear processes. It was found that PSD can be used for quantitative determination of process versus frequency. Mishra et al. [24] comprehensively analyzed the roughness characterization of the machined surface at different tool overhangs. Khana et al. [25] researched the effects of feed and vibrations on surface roughness with the use of power spectral analysis. Duparre et al. [26] compared the surface roughness measured with six different instruments and measurement techniques by using the RMS roughness which was calculated from APSD functions. Peng and Kirk [27] compared the fast Fourier transform (FFT) plots and angular spectrum of different types of wear particles. It was demonstrated that the angular spectrum which is calculated from APSD can be used to study the angular position of the surface profile to the measurement coordinate. Dong and Stout [28] comprehensively described the procedure of implementing APSD with a two-dimensional FFT algorithm and presented some sampling considerations to obtain proper APSDs. Wu et al. [29] applied Fourier transforms and power spectral analysis to the characterization of articular cartilage surface. Angular spectrum was also employed to judge the isotropy and anisotropy of the surface. Cheung and Lee [30] employed the power spectral method to analyze surface roughness profiles in single-point diamond turning. They also researched the effect of feed rate, corner radius, vibration, tool interference, and material swelling on the power spectrum.

A mass of researches on the relationship between cutting parameters and amplitude parameters of machined surface has been conducted. However, fewer researches on surface roughness and machining efficiency with respect to the relationship between *f_z_* and *a_e_* have been undertaken. Furthermore, PSD has been used to compare the properties of the surface generated with different machining methods, and to research the effect of cutting parameters and dynamic behavior during machining on the machined surface based on experiments. However, fewer studies have been conducted on the influence of the relationship between *f_z_* and *a_e_* on the PSD of machined surface. In this research, the evaluation of surface amplitude parameters and PSD was carried out based on a mathematical model of the end-milled surface topography. First, a mathematical model which can simulate surface topography in end milling was introduced and assessed by experiment. Second, the effects of *p* and *r* on amplitude parameters (*S_ba_* and *S_q_*) were investigated using the surface topography model. Third, the 1D PSD was calculated along both feed and step-over direction. The effect of *p* and *r* on the 1D PSD profile was researched. Finally, the angular spectrum was employed to analyze the distribution of PSD in different spatial directions of the machined surface.

## 2. Simulation Model of 3D Surface in End Milling

The surface topography model used in this research is based on our previous work and detailed introduced by Wang et al. ([31]). The modelling objective of this research is a round indexable insert (RDHW 10T3MO-MD04, Seco Company, Fagersta, Sweden) whose diameter is 10 mm. When the insert is fixed on the cutting tool, the axial rake angle is 4°.

### 2.1. Modelling of Cutting Tool Insert Trajectory

**Figure 1:**
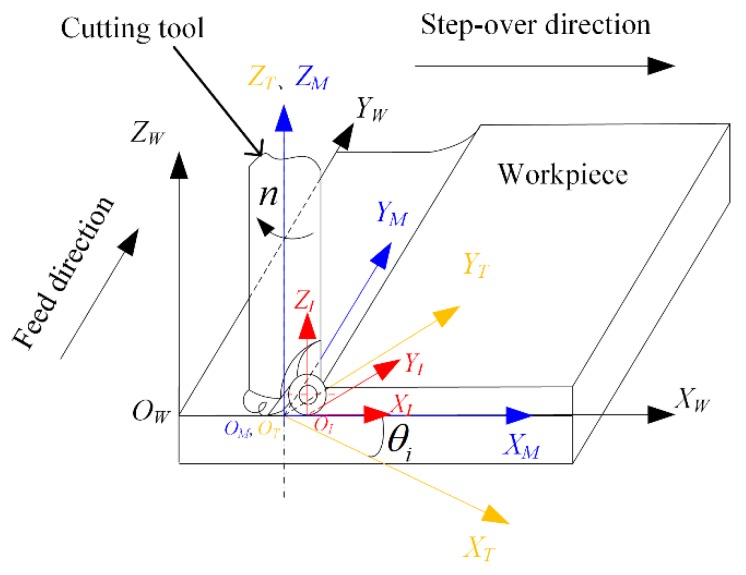
The machining coordinate systems used for the model.

In order to describe the relative trajectory between cutting tool and workpiece, the machining coordinate systems were established as shown in Figure 1.

The cutting insert coordinate system *O_I_-X_I_Y_I_Z_I_* is fixed on the cutting insert. The origin is the lowest point on the rake face. *X_I_* axis is perpendicular to the cutting tool axis. *Z_I_* axis is the ligature of the origin and the center of rake face and the positive direction of *Z_I_* axis is upward.

The machine tool spindle coordinate system *O_M_*-*X_M_Y_M_Z_M_* is fixed on the cutting tool and moves together with the feed motion. The origin is on the axis of the cutting tool. The *Y_M_* axis directs to the feed direction and the *Z_M_* axis directs upward.

The cutting tool coordinate system *O_T_*-*X_T_Y_T_Z_T_* is on the cutting tool and rotates with the rotation of the spindle. The origin *O_T_* coincides with *O_M_* if no vibration occurs on the spindle. The *X_T_* axis is parallel to the *X_I_* axis and the *Z_T_* axis is along the axis of the cutting tool.

The workpiece coordinate system *O_W_*-*X_W_Y_W_Z_W_* is set on the original machining point of the workpiece. The *X_W_Y_W_* plane is parallel to the work surface to be processed. The *X_W_* axis is along the step-over direction and *Y_W_* is along the feed direction.

After the machining coordinate systems were established, the motion trajectory of the arbitrary point *Q* on the cutting edge was derived by coordinate transformations in sequence as *O_I_-X_I_Y_I_Z_I_* to *O_T_*-*X_T_Y_T_Z_T_*, *O_T_*-*X_T_Y_T_Z_T_* to *O_M_*-*X_M_Y_M_Z_M_*, *O_M_*-*X_M_Y_M_Z_M_* to *O_W_*-*X_W_Y_W_Z_W_*. The kinematic equation of the cutting edge is as follows:

(1) $$\left\{ \begin{array}{l} x_{W}=R(1+\sin\alpha )\cos(\varphi_{i,1}+\omega t)+R(1-\cos\alpha )\sin(4{}^{\circ})\sin(\varphi_{i,1}+\omega t)+(i-1)a_{e}\\ y_{W}=-R(1+\sin\alpha )\sin(\varphi_{i,1}+\omega t)+R(1-\cos\alpha )\sin(4{}^{\circ})\cos(\varphi_{i,1}+\omega t)+v_{f}t\\ z_{W}=R(1-\cos\alpha )\cos(4{}^{\circ}) \end{array}\right.$$

where *R* is the radius of the cutting insert, *α* is the angle between the *Z_I_* axis and the ligature between *Q* and the center of the rake face, *ω* is the angular speed of the cutting tool, *i* is the number of feed motion, *t* is the cutting time from the beginning of the *i*_th_ feed motion until now, *φ_i,_*_1_ is the initial cutting angle, *a_e_* is the radial depth of cut, and *v_f_* is the feed speed.

After the kinematic equation of the cutting edge was derived, the algorithm presented by Wang et al. ([31]) was used to generate the surface topography in end milling. The whole algorithm was realized by using MATLAB software (MATLAB 7.13, MathWorks, Natick, MA, USA).

### 2.2. Experimental Verification of the Model

A validating experiment was conducted to assess the simulation model. The cutting conditions employed in the experiment are shown in Table 1. The axial depth of cut *a_p_*, cutting speed *v_c_*, *f_z_* and *a_e_*, were introduced as design variables. The experiment was conducted on AISI H13 steel whose hardness is 50 ± 1 HRC after hardening and high temperature tempering heat treatment.

A numerical control vertical machining center (YCM-V116B, Yongjin Machinery Co. LTD., Taiwan, China) was used to carry out the experiments. The maximum spindle speed of the machining center was 6000 r/min. One insert was employed and for every trial, a new insert was used to minimize the effect of tool wear. Dry machining and climb milling method were adopted.

The machined surfaces were observed by a white light interferometer (WYKO NT9300, Veeco Instruments Inc., Plainview, NY, USA). 3D arithmetic average deviation *S_ba_* and 3D root mean square deviation *S_q_* were employed so that the effect of the anisotropy characteristic of the milling surface could be taken into account. *S_ba_* and *S_q_* can be calculated as follows.

(2) $$S_{ba}=\frac{1}{MN}\sum_{i=1}^{M}\sum_{j=1}^{N}\left|Z_{ij}\right|$$

(3) $$S_{q}=\sqrt{\frac{1}{MN}\sum_{i=1}^{M}\sum_{j=1}^{N}{\left(Z_{ij}\right)}^{2}}$$

where *M* and *N* are the sampled data number in the sampling area along the feed and step-over directions, respectively. *Z* is the distance from the sampling points to the mean plane.

**Figure 2:**
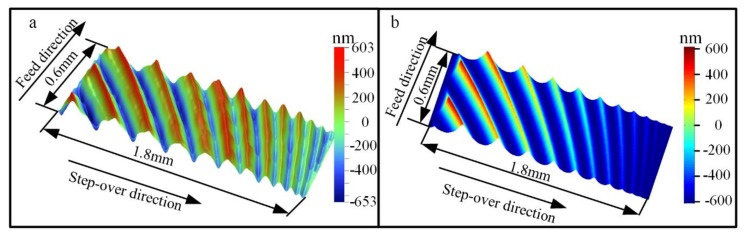
Comparison of surface topography (*v_c_* = 250 m/min, *f_z_* = 0.3 mm/z, *a_e_* = 1.8 mm, *a_p_* = 0.2 mm): (**a**) Experimental result; (**b**) simulated result.

The comparison of 3D surface topography between simulation and experiment is shown in Figure 2. The comparison result indicates that the experimental and simulated surface topography show great consistency.

The simulated and experimental results are listed in Table 2, which shows that the relative error ranges of *S_ba_* and *S_q_* between simulation and experiment are 3.23%~12.99% and 2.75%~6.56%, respectively. In other word, the simulation model was efficient enough to predict the surface roughness for the cutting condition in this research.

## 3. Results and Discussion

### 3.1. Influence of p and r on Amplitude Parameters

To research the influence of *p* and *r* on amplitude parameters, the developed model in Section 2 was used to conduct a simulating trial. The design of the trial is shown in Table 3.

**Figure 3:**
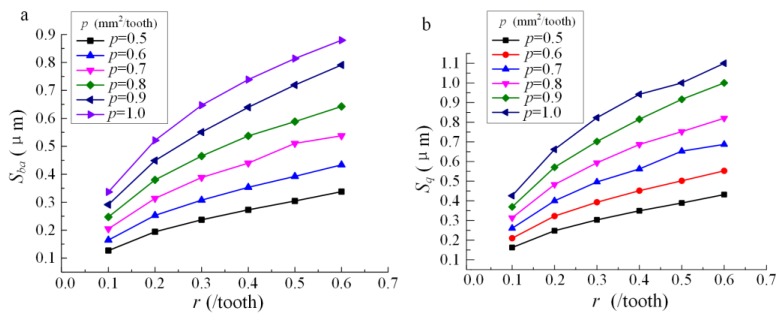
Profile of surface roughness under different *p* and *r*: (**a**) Profile of *S_ba_*; (**b**) profile of *S_q_*.

The simulation results are shown in Table 3 and Table 4. The influence of *r* and *p* on the amplitude parameters (*S_ba_* and *S_q_*) is shown in Figure 3. Different to the research result of the ball-end milling condition [17], which illustrates that the profile of amplitude parameters versus *r* is like a ‘check function’, the surface roughness monotonously increases with the increase of *r* and *p* for the machining condition in this research.

According to Figure 3, the profiles of *S_ba_* and *S_q_* versus *r* and *p* are similar to the exponential function. The function of surface roughness versus *r* and *p* can be expressed as:

(4) $$S_{}=Ar^{B}p^{C}$$

where *S* is the surface roughness, *A* is the constant coefficient of the exponential function. *B* and *C* are the exponential coefficients of the variables *r* and *p*, respectively.

Regression analysis was carried out by using the data in Table 3 and Table 4 for *S_ba_* and *S_q_*, respectively. The regression model of *S_ba_* and *S_q_* can be expressed as follows.

(5) $$S_{ba}=1.202r^{0.523}p^{1.46}$$

(6) $$S_{q}=1.523r^{0.538}p^{1.41}$$

Analysis of Variance (ANOVA) was employed to carry out the significance test for the regression models. The results of ANOVA are shown in Table 5 and Table 6. The value of *P* for the model is far less than 0.05. Thus, the relationships between amplitude parameters and the variables (*p* and *r*) are significant, in other word, the model is highly significant.

### 3.2. Research on PSD Based on the Simulation Model

The 3D surface evaluation parameters (*S_ba_* and *S_q_*) researched in Section 3.1 can be used to evaluate the characteristic of the machined surface along the normal direction of the machined surface. However, the lateral information of the machined surface cannot be analyzed. In addition, the anisotropy of the milled surface is also neglected in the amplitude parameters. So, in order to comprehensively analyze the milled surface topography, PSD was employed to describe the spatial frequency spectrum of the machined surface.

#### 3.2.1. PSD of the Machined Surface

1D PSD is used to analyze the frequency-domain characteristics of a surface profile along a specified direction (e.g., feed and step-over direction). The definition of the 1D PSD for a continuous surface profile *z*(*x*) is represented as follows:

(7) $$Z(f_{x})=\underset{T_{x}\to \infty }{\lim}\frac{1}{T_{x}}{\left|\int_{-\infty }^{\infty }z(x)\exp(-j2\pi xf_{x})dx\right|}^{2}$$

where *Z*(*f_x_*) is the PSD of *z*(*x*), *f_x_* is the frequency, and *T_x_* is the length of the surface profile.

In practice, the machined surface is usually obtained by a digitizing method with equal sampling intervals △*x* and fixed number (*N_x_*) of sampling points. So, PSD should be transformed into a discrete form as follows:

(8) $$Z(f_{p})=\frac{\Delta x}{N_{x}}{\left|\sum_{k=0}^{N_{x}-1}z(x_{k})\exp(-j2\pi pk/N_{x})\right|}^{2}$$

where *p* = 0, 1, 2,…, *N_x_* − 1, *f_p_* = *p*/(*N_x_*△*x*).

The research of 1D PSD depends on the direction of analysis, however, in order to research the whole frequency information of a 3D machined surface, APSD should be employed. Analogously to the derivation of 1D PSD, the APSD of a machined surface can be evaluated as follows:

(9) $$Z(f_{p},f_{q})=\frac{\Delta x\Delta y}{N_{x}N_{y}}{\left|\sum_{l=1}^{N_{x}-1}\sum_{k=1}^{N_{y}-1}z(x_{k},y_{l})\exp[-j2\pi (pk/N_{x}+ql/N_{y})]\right|}^{2}$$

where *N_x_* and *N_y_* are the sampled data numbers along x and *y* direction, respectively, *p* = 0, 1, 2,…, *N_x_* − 1, *q* = 0, 1, 2,…, *N_y_* − 1, △*x* and △*y* are the sampling interval along the *x* and *y* direction, *f_p_* = *p*/(*N_x_*△*x*), *f_q_* = *q*/(*N_y_*△*y*).

In addition, the angular spectrum which can be used to research the distribution of APSD in different spatial directions was also employed. For the calculation of the angular spectrum, APSD *Z*(*f_p_*,*f_q_*) in the Cartesian coordinate is first transferred into *Z*(*f_r_*,*θ*) in the polar coordinate, and then the angular spectrum can be calculated as follows.

(10) $$\begin{array}{l} S(\theta )=\int_{0}^{\frac{1}{2\sqrt{{\left(\Delta x\cos\theta \right)}^{2}+{\left(\Delta y\sin\theta \right)}^{2}}}}Z(f_{r},\theta )df_{r}\\ 0{}^{\circ}\leq \theta \leq 179{}^{\circ} \end{array}$$

#### 3.2.2. Influence of *p* and *r* on 1D PSD

In order to research the influence of *p* and *r* on 1D PSD, the developed model in Section 2 was used to conduct a simulating trial. The design of the trial is shown in Table 7. The surface profiles along feed direction (*Y* direction) and step-over direction (*X* direction) were extracted from the simulated surface topography and used to analyze the PSD. For each direction, 40 profiles at different locations were extracted. Then the average of the calculated PSD was used for the final result.

**Figure 4:**
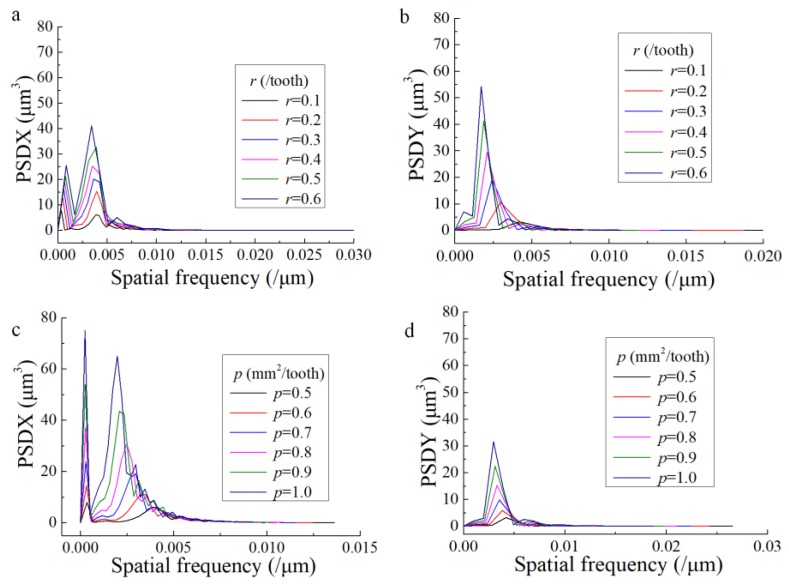
Effects of *r* and *p* on 1D power spectral density (PSD) along different directions (**a**) Effect of *r* on 1D PSD along step-over direction; (**b**) Effect of *r* on 1D PSD along feed direction; (**c**) Effect of *p* on 1D PSD along step-over direction; (**d**) Effect of *p* on 1D PSD along feed direction.

The effect of *p* and *r* on the 1D PSD profile is shown in Figure 4. It can be seen that PSD is concentrated in several ranges of frequencies. For the PSD profile along the step-over direction, there exist two peaks, whereas only one obvious peak is found for that along the feed direction. Figure 4 also describes that the PSD amplitude is higher with higher values of both *p* and *r* along the feed and step-over directions. This complies with the conclusion in Section 3.1 that the amplitude parameters monotonously increases with the increase of *r* and *p*.

The frequencies corresponding to the peaks of the PSD profiles were extracted and the wavelengths corresponding to these frequencies are shown in Table 8, in which *X_p_*_1_ and *X_p_*_2_ are the wavelengths for the first and second peaks of the PSD profile along the step-over direction, *Y_p_* is the wavelength for the peak of the PSD profile along the feed direction.

It can be found from Table 8 that *X_p1_* values for all the trials are approximately 1.28 times of the values of *a_e_* which means that the effect of *a_e_* on the surface profile along the step-over direction is significant. In addition, *Y_p_* values for all the trials are approximately 1.07 times of the values of *f_z_* which means *f_z_* is the most significant factor that influences the surface contour along the feed direction. Furthermore, it is interesting that the value of *X_p2_* barely changes with the increase of *r* whereas it shows an increasing tendency when *p* increases. Obviously, when the value of *p* is constant, *f_z_* increases and *a_e_* decreases with the increase of *r*. This may be the reason why the value of *X_p2_* remains quasi-constant with the increase of *r*. In another words, the second peak of the PSD profile along the step-over direction is the result of the common influence of *f_z_* and *a_e_*.

#### 3.2.3. Research on APSD and the Angular Spectrum of the Milled Surface

**Figure 5:**
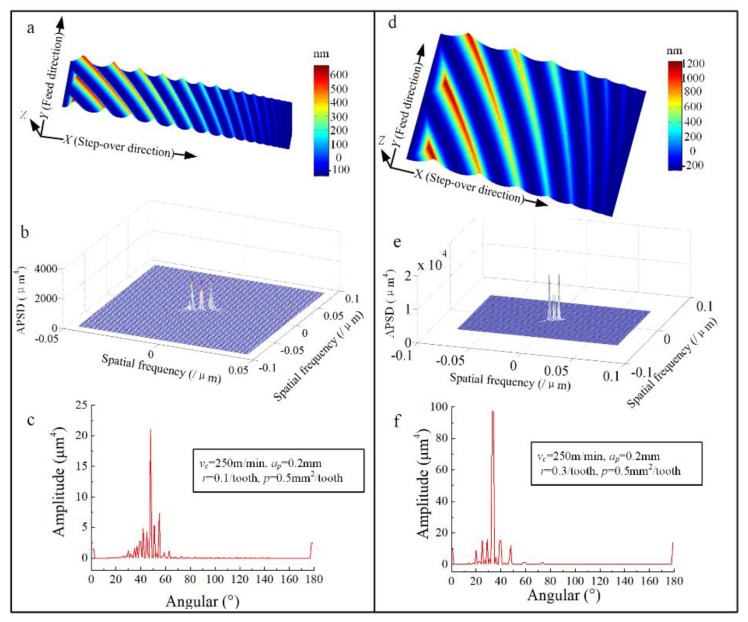
Three-dimensional surface topography and its area power spectral density (APSD) and angular spectrum. (**a**,**d**) Three dimensional surface topography; (**b**,**e**) APSD of the surface; (**c**,**f**) Angular spectrum.

The APSD and angular spectrum of all the trials in Table 7 were calculated and the results of two example trials are presented in Figure 5. It can be seen from the APSD that the amplitudes concentrate at several prominent frequencies and the surface texture distributes along specific directions. The angular spectrum can indicate the angle position of spectral intensity to the *X* axis in the *X*–*Y* plane. The angular spectrum profile in “Figure 5c,f” shows several peaks at the corresponding angles. It can be found that the number of the angle spectrum peaks for the specific machined surface approximately equals the number of residual ridges on the surface topography in “Figure 5a,d”. This is because the contour map of APSD is perpendicular to the surface texture direction [20]. So, for every ridge of the machined surface, a spectral energy distribution exists along the direction perpendicular to the ridge orientation.

**Figure 6:**
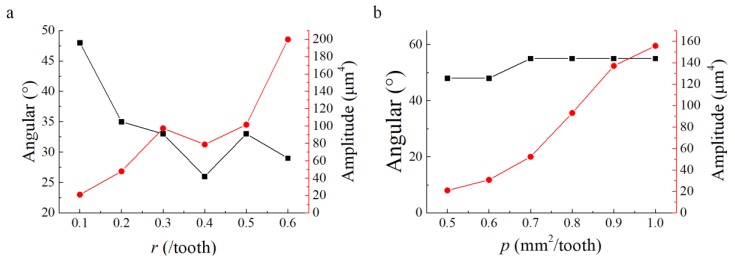
Effect of *p* and *r* on the peak amplitudes of the angular spectrum and the corresponding angular. (**a**) Effect of *r*; (**b**) Effect of *p*.

Furthermore, it also can be seen from Figure 5c,f that for each angular spectrum, there exists a maximum peak. The effect of *p* and *r* on the maximum peak amplitude and the corresponding angle is shown in Figure 6. The result shows that the maximum amplitude of the energy spectrum has a tendency to increase when *r* increases, while the corresponding angle tends to decrease. With the increase of *p* value, the maximum amplitude of the energy spectrum tends to increase, while the corresponding angle barely changes. In other words, the effect of *p* on the distribution direction of the surface energy spectrum is not significant. The main factor influencing the direction of surface energy spectrum distribution is the value of *r*.

## 4. Conclusions

Based on a surface topography model, the amplitude surface roughness (*S_ba_* and *S_q_*) and PSD of the end milled surface were calculated. The effects of *p* and *r* on *S_ba_*, *S_q_* and PSD were also investigated. The conclusions derived from the research can be summarized as follows:

- *S_ba_* and *S_q_* have a monotonous tendency to increase with the increase of *r* and *p*. The exponential models of *S_ba_* and *S_q_* versus *r* and *p* were fitted.
- The effects of *p* and *r* on the 1D PSD profile along the feed direction and step-over direction were researched. The result shows that the PSD amplitude is higher with higher values of both *p* and *r* along the feed and step-over directions. *f_z_* affects the peaks of the 1D PSD along both directions whereas *a_e_* affects the peak of the 1D PSD along the step-over direction.
- The angular spectrum of the surface was calculated by the APSD. It can be found that the number of the angle spectrum peaks for the specific machined surface approximately equals the number of residual ridges on the surface topography. The research on the effect of *p* and *r* on the angular spectrum reveals that *r* is the dominant factor influencing the direction of surface energy spectrum distribution.

## Figures and Tables

**Table 1:** Cutting conditions of the experiment.

No.	*v_c_* (m/min)	*f_z_* (mm/tooth)	*a_e_* (mm)	*a_p_* (mm)
1	250	0.3	1.8	0.2
2	190	0.25	1.8	0.5
3	220	0.35	0.6	0.8
4	160	0.35	1.2	1.4

**Table 2:** Results of the validation experiment.

No.	Surface Roughness	Experimental Result (μm)	Simulated Result (μm)	Error (%) ^1^
1	*S_ba_*	0.215	0.194	9.77
	*S_q_*	0.255	0.248	2.75
2	*S_ba_*	0.154	0.134	12.99
	*S_q_*	0.183	0.171	6.56
3	*S_ba_*	0.101	0.0904	10.5
	*S_q_*	0.124	0.116	6.45
4	*S_ba_*	0.186	0.180	3.23
	*S_q_*	0.240	0.231	3.75

^1^ Error = (Experimental result – Simulation result)/Experimental result.

**Table 3:** *S_ba_* (μm) for different values of *p* (mm^2^/tooth) and *r* (/tooth).

	*p*	0.5	0.6	0.7	0.8	0.9	1
*r*							
0.1		0.127	0.165	0.205	0.247	0.291	0.337
0.2		0.194	0.253	0.314	0.38	0.449	0.521
0.3		0.237	0.307	0.389	0.465	0.551	0.647
0.4		0.273	0.353	0.44	0.537	0.64	0.739
0.5		0.304	0.392	0.51	0.588	0.719	0.814
0.6		0.338	0.434	0.538	0.643	0.791	0.879

**Table 4:** *S_q_* (μm) for different values of *p* (mm^2^/tooth) and *r* (/tooth).

	*p*	0.5	0.6	0.7	0.8	0.9	1
*r*							
0.1		0.162	0.21	0.26	0.314	0.369	0.426
0.2		0.248	0.322	0.4	0.483	0.571	0.662
0.3		0.303	0.393	0.496	0.594	0.702	0.823
0.4		0.349	0.452	0.562	0.687	0.815	0.942
0.5		0.389	0.502	0.653	0.752	0.917	1
0.6		0.432	0.553	0.687	0.82	1	1.1

**Table 5:** ANOVA of the regression model for *S_ba_*.

Model	Degree of Freedom	Sum of Squares	Mean Square	*F* Value	*P*
Regression	2	7.8912	3.9456	7201.76	0.000
Residual	33	0.0181	0.0005		
Total	35	7.9092	*R*^2^: 0.998, *R*^2^(*Adj.*): 0.998		

**Table 6:** ANOVA of the regression model for *S_q_*.

Model	Degree of Freedom	Sum of Squares	Mean Square	*F* Value	*P*
Regression	2	7.8022	3.9011	5719.82	0.000
Residual	33	0.0225	0.0007		
Total	35	7.8247	*R*^2^: 0.997, *R*^2^(*Adj.*): 0.997		

**Table 7:** Design of the simulating trials (*v_c_* = 250 m/min, *a_p_* = 0.2 mm).

No.	1	2	3	4	5	6	7	8	9	10	11
*r* (/tooth)	0.1	0.2	0.3	0.4	0.5	0.6	0.1	0.1	0.1	0.1	0.1
*p* (mm^2^/tooth)	0.5	0.5	0.5	0.5	0.5	0.5	0.6	0.7	0.8	0.9	1

**Table 8:** Wavelength corresponding to the peaks of the power spectral density (PSD) profile (*v_c_* = 250 m/min, *a_p_* = 0.2 mm).

No.	*R* (/tooth)	*P* (mm^2^/tooth)	*f_z_* (mm/tooth)	*a_e_* (mm)	*X_p1_* (mm)	*X_p2_* (mm)	*Y_p_* (mm)
1	0.1	0.5	0.224	2.236	2.862	0.26	0.239
2	0.2	0.5	0.316	1.581	2.024	0.253	0.337
3	0.3	0.5	0.387	1.291	1.653	0.275	0.413
4	0.4	0.5	0.447	1.118	1.431	0.286	0.477
5	0.5	0.5	0.500	1.000	1.28	0.256	0.533
6	0.6	0.5	0.548	0.913	1.169	0.292	0.584
7	0.1	0.6	0.245	2.449	3.135	0.285	0.261
8	0.1	0.7	0.265	2.646	3.387	0.339	0.282
9	0.1	0.8	0.283	2.828	3.62	0.402	0.302
10	0.1	0.9	0.300	3.000	3.84	0.48	0.32
11	0.1	1	0.316	3.162	4.048	0.506	0.337
